# Phosphate Homeostasis − A Vital Metabolic Equilibrium Maintained Through the INPHORS Signaling Pathway

**DOI:** 10.3389/fmicb.2020.01367

**Published:** 2020-07-14

**Authors:** Sisley Austin, Andreas Mayer

**Affiliations:** Département de Biochimie, Université de Lausanne, Lausanne, Switzerland

**Keywords:** nutrient signaling, phosphate, acidocalcisome, SPX, polyphosphate, inositol pyrophosphate, Tor, PKA

## Abstract

Cells face major changes in demand for and supply of inorganic phosphate (P_i_). P_i_ is often a limiting nutrient in the environment, particularly for plants and microorganisms. At the same time, the need for phosphate varies, establishing conflicts of goals. Cells experience strong peaks of P_i_ demand, e.g., during the S-phase, when DNA, a highly abundant and phosphate-rich compound, is duplicated. While cells must satisfy these P_i_ demands, they must safeguard themselves against an excess of P_i_ in the cytosol. This is necessary because P_i_ is a product of all nucleotide-hydrolyzing reactions. An accumulation of P_i_ shifts the equilibria of these reactions and reduces the free energy that they can provide to drive endergonic metabolic reactions. Thus, while P_i_ starvation may simply retard growth and division, an elevated cytosolic P_i_ concentration is potentially dangerous for cells because it might stall metabolism. Accordingly, the consequences of perturbed cellular P_i_ homeostasis are severe. In eukaryotes, they range from lethality in microorganisms such as yeast (Sethuraman et al., 2001; Hürlimann, 2009), severe growth retardation and dwarfism in plants (Puga et al., 2014; Liu et al., 2015; Wild et al., 2016) to neurodegeneration or renal Fanconi syndrome in humans (Legati et al., 2015; Ansermet et al., 2017). Intracellular P_i_ homeostasis is thus not only a fundamental topic of cell biology but also of growing interest for medicine and agriculture.

## Phosphate Control Is a Challenge for Cells

Cells should coordinate their systems for the uptake, export, and storage of P_i_ in order to strike a delicate balance between the biosynthetic requirements for P_i_ and the risks of an excessive cytoplasmic P_i_ concentration. To achieve this, they may use signaling networks that sense extra- and intracellular P_i_ and a buffering system for cytosolic P_i_. In this review, we do not provide a global overview of phosphate homeostasis in all model organisms studied in this respect because this adds significant complexity and detail, resulting for example from multicellularity, tissue differentiation, or the complexity of their life cycles. We will rather focus on the yeast *Saccharomyces cerevisiae* because it is a unicellular model and the eukaryotic model system in which relevant pathways and mechanisms have been best characterized. Focusing on a single well-characterized and simple model provides the best basis for our effort to develop a conceptual framework for intracellular phosphate homeostasis, which may provide leads to dissect this crucial homeostatic system also in other eukaryotic organisms (Table 1). We therefore add information from other models only where it is necessary to provide information that is not available for *S. cerevisiae*.

**TABLE 1:** Proteins and terms repeatedly used in the review.

Arg82	Inositol polyphosphate multikinase (IPMK); sequentially phosphorylates InsP_3_ to form InsP_5_
Ddp1	Member of the Nudix hydrolase family; displays di-phosphoinositol polyphosphate hydrolase activity; hydrolyzes the ß-phosphates of InsP_8_ and InsP_7_
Gde1	Glycerophosphocholine (GroPCho) phosphodiesterase; carries an SPX domain
INPHORS	Acronym for intracellular phosphate reception and signaling
InsPP	Inositol pyrophosphate
Ipk1	Nuclear inositol pentakisphosphate 2-kinase; converts InsP_5_ to InsP_6_
Kcs1	Inositol hexakisphosphate kinase; phosphorylates InsP_6_ or Ins(1,3,4,5,6)P_5_, creating 5-InsP_7_ or 5PP-InsP_4_, respectively
Phm8	Lysophosphatidic acid phosphatase
PHO pathway	Phosphate-responsive signaling pathway regulating transcription in *Saccharomyces cerevisiae*
Pho11	Cell wall-associated acid phosphatase
Pho12	Cell wall-associated acid phosphatase
Pho2	Transcription factor for the PHO pathway; cooperates with Pho4
Pho4	Transcription factor for the PHO pathway; cooperates with Pho2
Pho5	Repressible secreted acid phosphatase
Pho8	Repressible alkaline phosphatase located in the vacuole
Pho80	Cyclin subunit of the cyclin-dependent Pho85/80/81 kinase
Pho81	Cyclin-dependent kinase inhibitor (CKI); regulatory subunit of the Pho85/80/81 kinase; possesses an SPX domain
Pho84	High-affinity inorganic phosphate plasma membrane transporter
Pho85	Catalytic subunit of the cyclin-dependent Pho85/80/81 kinase; Pho85 associates with at least 10 different cyclins to regulate a wide spectrum of target proteins involved in many cellular processes
Pho87	Low-affinity inorganic phosphate plasma membrane transporter; carries an SPX domain
Pho89	High-affinity inorganic phosphate plasma membrane transporter
Pho90	Low-affinity inorganic phosphate plasma membrane transporter; carries an SPX domain
Pho91	Low-affinity inorganic phosphate transporter in the vacuolar membrane; possesses an SPX domain; homologs are rice OsSPX-MFS3 and *Trypanosoma brucei* TbPho91
Pho92	Posttranscriptional regulator of phosphate metabolism; regulates the degradation of Pho4 mRNA by binding to its 3’-UTR in a P_i_-dependent manner
P_i_	Inorganic phosphate
PKA	cAMP-dependent protein kinase; controls a variety of cellular processes, including metabolism
Plc1	Phospholipase C; hydrolyzes phosphatidylinositol 4,5-biphosphate (PIP_2_) to generate the signaling molecules InsP_3_ and 1,2-diacylglycerol (DAG)
polyP	Polymer of up to a thousand P_i_ units linked through phosphoric anhydride bonds
Ppn1	Endo- and exopolyphosphatase in vacuoles
Ppn2	Zn^2+^-dependent endopolyphosphatase in vacuoles
Ppx1	Soluble exopolyphosphatase in the cytosol
Rim15	Serine/threonine protein kinase; regulates cell proliferation in response to nutrients
Siw14	Member of the dual-specificity phosphatase family; hydrolyzes the ß-phosphates of 5-InsP_7_ and InsP_8_
Spl2	Regulator of low-affinity phosphate transporter
SPX domain	Domain binding inositol pyrophosphates and Pi; involved in the regulation of phosphate homeostasis
Syg1	Plasma membrane protein presumed to export inorganic phosphate; possesses an SPX domain; similarities with human XPR1
Vip1	Diphosphoinositol pentakisphosphate kinases (PPIP5K); contains both a kinase and a histidine acid phosphatase domain; the kinase domain phosphorylates InsP_6_ and 5-InsP_7_ to generate 1-InsP_7_ and 1,5-InsP_8_, respectively
VTC complex	Polyphosphate polymerase complex; synthesizes polyP from nucleotide triphosphates and translocates it across the vacuolar membrane; composed of four subunits: Vtc4, the catalytically active subunit; Vtc1/2 or Vtc1/3, mainly localized in the ER or in vacuoles, respectively; and the regulatory subunit Vtc5. Vtc2, Vtc3, Vtc4, and Vtc5 possess an SPX domain

## Strategies for Phosphate Homeostasis

Multicellular organisms can regulate P_i_ concentration at the organismal level. Humans, for example, maintain P_i_ concentration in circulating body fluids through filtration at the level of the kidneys and controlled reabsorption (Biber et al., 2013; Sabbagh, 2013). They can access the apatite in bones as a huge P_i_ reserve. These well-studied mechanisms provide the individual cells with a relatively constant environment of extracellular P_i_, alleviating the need for complex P_i_-foraging programs at the cellular level. Nevertheless, human tissues widely express a regulated P_i_ exporter in the plasma membrane (XPR1) (Giovannini et al., 2013), which suggests that they might also maintain a safeguard against peaks of cytosolic P_i_. Dysregulation of XPR1 leads to neurodegeneration and brain calcification, suggesting that XPR1 might dampen the significant changes of P_i_ concentration in brain cells that can occur depending on its metabolic state (McIlwain et al., 1951). Interestingly, XPR1 is even important for P_i_ homeostasis at the organismal level because it is implicated in the reabsorption of P_i_ across the renal tubules of the kidney (Ansermet et al., 2017).

Whereas P_i_ regulation at the organismal level has been intensely studied, particularly in humans (Biber et al., 2013; Sabbagh, 2013), it is poorly understood how cytosolic P_i_ concentration is measured and regulated at the level of individual cells. Unicellular organisms can experience rapid changes in P_i_ availability. They use multiple systems to maintain P_i_ homeostasis, which allow the cell to mount a graded response that is tuned to the degree of P_i_ availability and consumption (Bostian et al., 1983). Cells respond to P_i_ scarcity with various foraging strategies (Conrad et al., 2014; Puga et al., 2017), in which they try to liberate P_i_ from a variety of extracellular substrates. They can express P_i_ importers of low and high affinity, which allow them to acquire P_i_ under a wide range of external concentrations. They maintain important P_i_ stores in acidocalcisome-like organelles, which, in case of sudden P_i_ starvation, can guarantee them sufficient reserves to finish the next cell cycle and make an ordered transition into a robust quiescent state. Finally, cells can also “recycle” and liberate P_i_ from internal sources, such as nucleotides and phospholipids. While such recycling appears senseless for a growing cell—because it will need those compounds to grow—it may become critical for cells that have already arrested growth. They may choose to reallocate their internal P_i_ pool in order to perform new biosyntheses that are critical to survive in the non-dividing, starved state. Since P_i_ scarcity retards growth but as such does not seem to be lethal, mounting starvation responses can rely on (relatively slow) transcriptional P_i_ starvation programs. But cells may also experience a sudden excess of P_i_ and may need highly reactive mechanisms to protect themselves against the potentially lethal consequences. In this situation, posttranslational signaling becomes important, allowing to rapidly and simultaneously regulate multiple systems for import, export, and storage of P_i_ and to thus dampen cytosolic P_i_ peaks.

## Acidocalcisomes: a Conserved Organelle With a Role in Phosphate Buffering?

**FIGURE 1:**
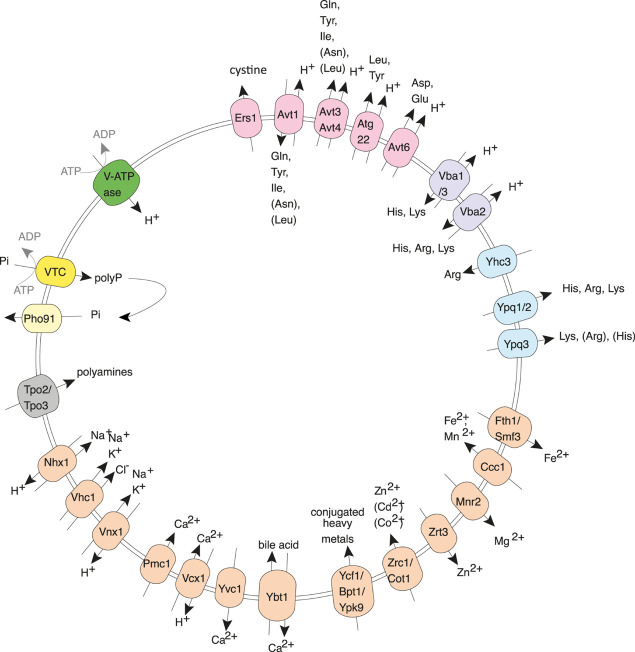
Yeast vacuoles as an example of an acidocalcisome-like organelle. The polyphosphate polymerase VTC transfers inorganic phosphate (P_i_) into vacuoles by converting it into polyphosphate (polyP) and translocating it across the membrane. Here, the lumenal polyphosphatases can convert polyP back into P_i_, which can be re-exported into the cytosol through Pho91. Apart from polyP, yeast vacuoles actively accumulate many other compounds that are typical of acidocalcisomes, such as Ca^2+^, Mn^2+^, Zn^2+^, and basic amino acids. Examples of the involved transporters are shown in the illustration.

Acidocalcisomes are membrane-bound organelles that are conserved from bacteria to mammals (Docampo and Huang, 2016), but their functions are poorly understood. Yeast cells have an acidocalcisome-like compartment, the vacuole, which carries transporters for all compounds that are typically concentrated in acidocalcisomes (Figure 1). Typical acidocalcisome features comprise high concentrations of divalent cations, an acidic pH, and several hundred millimolars of the basic amino acids arginine as lysine. P_i_ is accumulated to similarly high concentrations in the form of polyphosphate (polyP), a polymer of up to a thousand P_i_ units linked through phosphoric anhydride bonds. PolyP is stored in the acidocalcisome lumen, where polyphosphatases are also located. It is assumed that these enzymes can hydrolyze polyP, which may allow re-export of the liberated P_i_ into the cytosol (Gerasimaite and Mayer, 2016, 2017). Thereby, acidocalcisome-like organelles might be important buffering devices for cytosolic P_i_. In line with this, cells lacking polyP show an accelerated activation of the transcriptional phosphate starvation response on low-P_i_ media (Neef and Kladde, 2003; Thomas and O’Shea, 2005). They also show delays in the S-phase and slower dNTP synthesis, probably because a rapid duplication of nucleic acids and phospholipids generates a P_i_ requirement that transiently exceeds the uptake capacity of the cell and necessitates the engagement of internal P_i_ reserves (Neef and Kladde, 2003; Bru et al., 2016). A major open question is whether and how acidocalcisome-like vacuoles behave in these situations, i.e., how the turnover and release of P_i_ back into the cytosol are triggered, such that futile cycles of polyP synthesis and hydrolysis are avoided. This is a pre-condition for acidocalcisome-like vacuoles to constitute an efficient regulated P_i_ buffer.

While acidocalcisomes store high concentrations of phosphate and are, hence, prime candidates for a P_i_-buffering system, it must be kept in mind that other organelles also use and/or liberate P_i_ as part of their metabolic functions. For example, the endoplasmic reticulum (ER) lumen contains many chaperones that hydrolyze ATP (Depaoli et al., 2019). The Golgi liberates P_i_ as a by-product of the glycosylation reactions in this compartment, and it is likely that this P_i_ is recycled back to the cytosol through a dedicated channel, Erd1 (Snyder et al., 2017). Likewise, the mitochondria permanently import large quantities of P_i_ in order to regenerate ATP from ADP (Palmieri and Monné, 2016). Phosphate homeostasis in the cytosol will be influenced by these different processes, but their impact has yet remained essentially unaddressed.

## Basic Setup for P_i_ Regulation in Yeast

**FIGURE 2:**
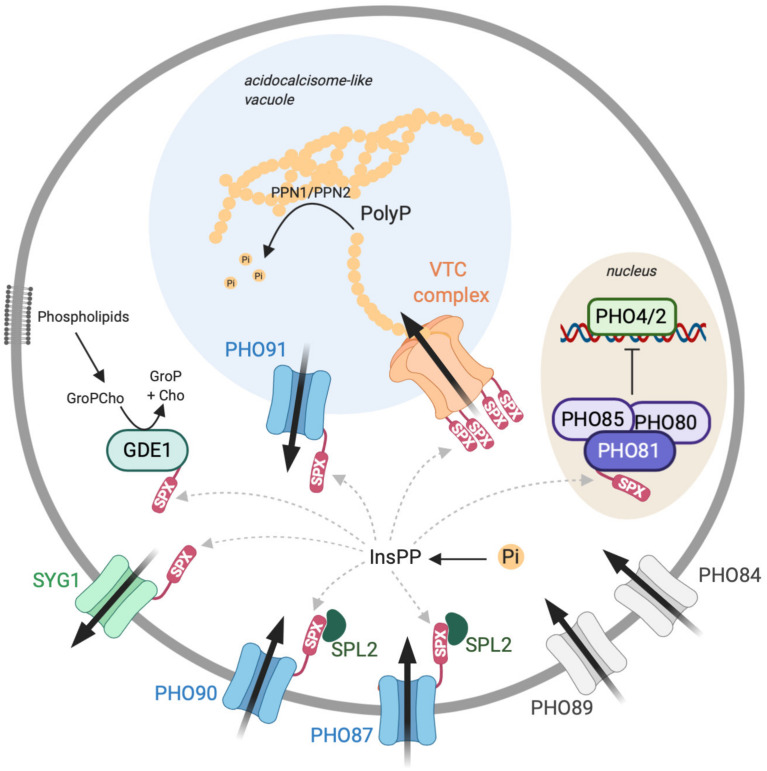
The INPHORS (intracellular phosphate reception and signaling) pathway for inorganic phosphate (P_i_) homeostasis in the cytosol, based on known components from *Saccharomyces cerevisiae*. P_i_ can be imported into the cytosol by high-affinity (*Pho89*/*84*) and low-affinity importers (*Pho87*/*90*) and by a P_i_-permeable vacuolar channel (*Pho91*). It can be exported from the cytosol by secretion into the extracellular space (*Syg1*) or by conversion into polyphosphate (polyP) and transferred into the acidocalcisome-like vacuole (*VTC*). Inside this organelle, polyphosphatases can reconvert polyP into P_i_ to make it available for export into the cytosol. A P_i_-regulated kinase (*Pho80*/*85*/*81*) phosphorylates transcription factors (*Pho4*/*2*) for the transcriptional phosphate starvation response. A sensor for cytosolic P_i_ might regulate the levels of inositol pyrophosphates (InsPPs; e.g., 5-InsP_7_), which then act on SPX domains. These should, when bound to InsPPs, inactivate proteins importing P_i_ and activate proteins exporting P_i_ from the cytosol.

P_i_ homeostasis can be achieved by controlling a variety of processes in a synergistic manner, such as the import and export of P_i_, its intracellular storage and re-mobilization, active foraging for P_i_ in the environment, and P_i_ recycling within the cell. In yeast, many components contributing to these processes are known. At least 25 out of the approximately 6,000 genes in yeast are directly implicated in P_i_ homeostasis, illustrating the crucial importance of this parameter for the cells. They comprise secreted acid phosphatases to liberate P_i_ from a variety of substrates in the environment (Oshima, 1997): P_i_ importers of high (Pho84 and Pho89) and low (Pho87 and Pho90) affinity and a putative P_i_ exporter in the plasma membrane (Syg1; Figure 2) (Persson et al., 2003). Cells can also recycle P_i_ from internal sources, e.g., by liberating it from nucleotides or phospholipids (Phm8 and Gde1) (Patton-Vogt, 2007; Xu et al., 2013). An ATP-driven protein complex (VTC complex) exists for storing and concentrating P_i_ as an osmotically inactive polyphosphate and depositing it inside the vacuole, from where it can be re-mobilized by polyphosphatases (Ppn1 and Ppn2) (Sethuraman et al., 2001; Gerasimaite and Mayer, 2016, 2017). The vacuolar membrane carries a P_i_ transporter (Pho91) (Hürlimann et al., 2009), which might participate in the re-mobilization of polyP and export P_i_ resulting from polyP hydrolysis into the cytoplasm.

Many genes involved in P_i_ homeostasis are expressed through a transcriptional control mechanism, the phosphate-responsive signal transduction (PHO) pathway (Yoshida et al., 1989b). The PHO pathway is regulated through a P_i_-responsive kinase (Pho80/Pho85/Pho81), which phosphorylates the transcription factor Pho4, thereby keeping it inactive. P_i_ starvation favors the dephosphorylation of Pho4 and allows it to activate a wide spectrum of genes dedicated not only to P_i_ import and storage but also to foraging for extracellular P_i_ and the recycling of intracellular phosphate (Carroll and O’Shea, 2002). The response is graded: Upon moderate phosphate starvation, high-affinity P_i_ importers (Pho84) and the polyP polymerase VTC are induced, whereas proteins for P_i_ foraging are upregulated only upon further P_i_ limitation (Vardi et al., 2014). This includes phosphatases that are secreted from the cells in order to recover P_i_ from phosphorylated substrates in their environment (Oshima, 1997). In addition, phosphate starvation leads to the enhanced production of proteins for P_i_ recycling, which allows the cell to recover P_i_ from internal molecules (Ogawa et al., 2000). Examples are the glycerophosphodiesterase Gde1 (Fisher et al., 2005; Patton-Vogt, 2007), which can remove P_i_ from intermediates of lipid metabolism, or Phm8, which dephosphorylates nucleoside monophosphates (Xu et al., 2013). For all of these systems, it has remained enigmatic how P_i_ availability is measured in order to regulate them.

Yeast cells thus use multiple systems and mechanisms to control cytosolic P_i_. We can expect that this results in a high degree of redundancy, which can be taken as an indication that P_i_ homeostasis is of critical importance for the cells. For the exploration of P_i_ homeostasis, this comes as a blessing and a curse at the same time: On the one hand, mutants in a single compound of this complex system will usually be viable and amenable to investigation, but, on the other hand, redundancy renders it more difficult to generate clear phenotypes that are necessary to analyze its function.

Cells should benefit from information about the concentrations of free P_i_ in their environment, within the cytoplasm, and within the subcellular compartments. How cells perceive or measure these important parameters is not well understood at this point. Available evidence suggests a transceptor for extracellular P_i_. A transceptor is a transporter that senses an external substrate independently of its transport activity. Pho84 was (together with the low-affinity transporter Pho87) proposed to sense extracellular P_i_ in this manner and to regulate intracellular responses, such as the protein kinase A (PKA) pathway (Giots et al., 2003; Mouillon and Persson, 2005, 2006; Popova et al., 2010). The transceptor does not appear to address all P_i_-regulated events because a major P_i_-dependent response, the transcriptional starvation response (PHO pathway), reacts to intracellular rather than extracellular P_i_, arguing against a general role of Pho84 or Pho87 in P_i_ sensing (Wykoff and O’Shea, 2001; Auesukaree et al., 2004; Desfougères et al., 2016). We thus face the possibility that cells may use at least two different signaling mechanisms that distinguish between cytosolic and extracellular P_i_. Such a differentiation might be useful because the PKA pathway is particularly important when cells exit a growth arrest resulting from nutrient limitation (Conrad et al., 2014). In this situation—of a non-dividing cell—cytosolic P_i_ is not a useful readout because there is no P_i_ consumption and, hence, the cytosolic concentration may easily be sufficient. The decision to reenter the cell cycle can more reliably be instructed by information about the external P_i_ supply, which will be necessary to support the next S-phase. Hence, the interest of a transceptor. In contrast, the measurement of cytosolic P_i_ is highly relevant in an actively growing and dividing cell because, here, the duplication of all cellular components requires the uptake of enormous amounts of P_i_. In this situation, the exhaustion of an existing external P_i_ resource usually occurs gradually, calling for the activation of additional P_i_ foraging, which is one of the main purposes of the phosphate starvation response. However, the consumption of cytosolic P_i_ changes drastically in the different phases of the cell cycle, being by far the highest in the S-phase. A dividing cell will therefore have to closely monitor and regulate its cytosolic P_i_ in order to avoid large changes in this critical parameter. The sensing mechanism for intracellular P_i_ should hence address multiple proteins for the mobilization, transport, and storage of P_i_ in order to stabilize the P_i_ concentration in the cytosol in the face of a grossly fluctuating demand.

## INPHORS: a Conserved Phosphate Signaling Pathway for Intracellular P_i_

A yeast cell thus uses multiple and potentially redundant systems to supply P_i_ to the cytoplasm or withdraw it from there. A key question is how the activities of these systems are coordinated. An important hint in this respect comes from the sequences of these proteins since many of them carry an SPX (*S*yg1/*P*ho81/*X*PR1) domain. The fact that all of the 10 yeast proteins that contain an SPX domain are involved in P_i_ homeostasis strongly suggests a role of this domain in coordinating P_i_ signaling (Secco et al., 2012a, b). SPX appears to act in conjunction with inositol pyrophosphates (InsPPs), molecules which are also critical for P_i_ homeostasis (Auesukaree et al., 2005; Wild et al., 2016).

**FIGURE 3:**
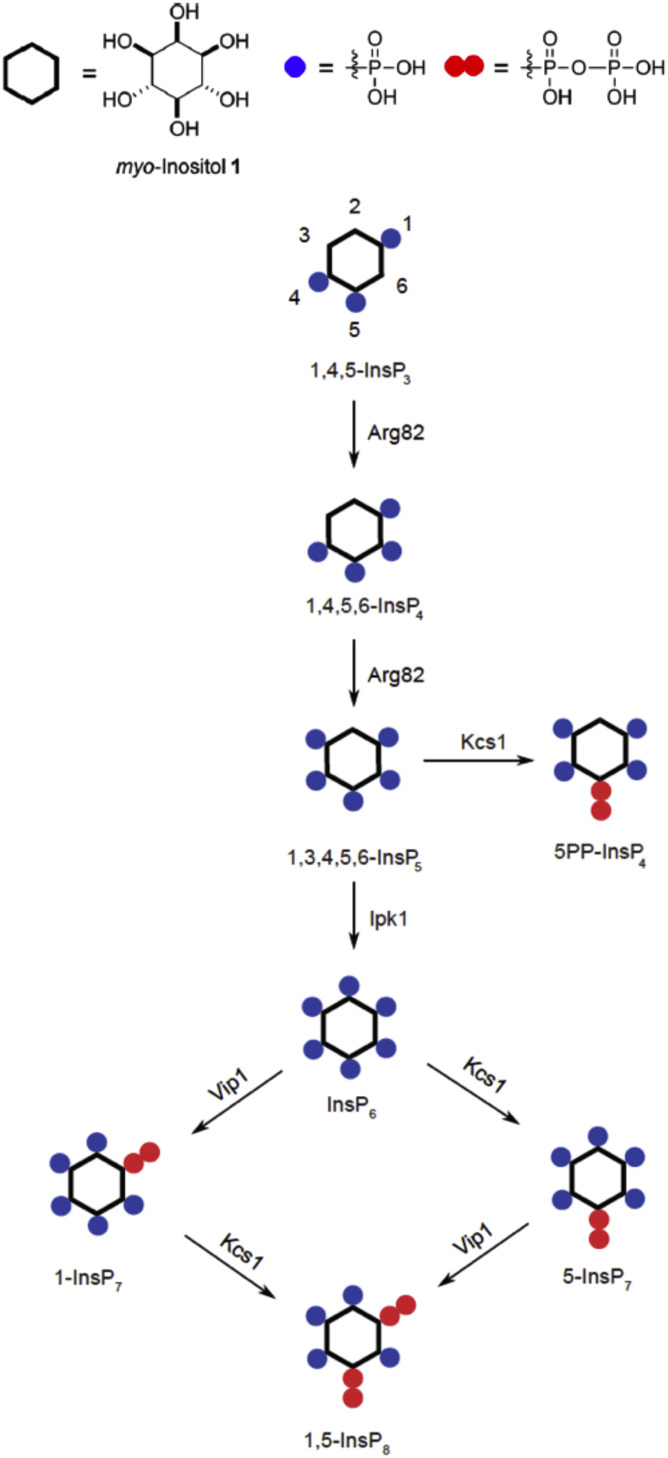
Synthesis of inositol pyrophosphates (InsPPs) in yeast cells, starting from InsP_3_. Responsible enzymes are indicated for each step. *Blue dots* indicate a phosphoryl group and *pairs of red dots* a pyrophosphoryl group on the inositol ring.

InsPPs are highly phosphorylated inositol species with at least one pyrophosphate moiety. Their abundance increases in correlation with the availability of P_i_ in the media (and hence presumably in response to the resulting changes in cytosolic P_i_) (Lee et al., 2007; Lonetti et al., 2011; Wild et al., 2016; Gu et al., 2017). They bind to the SPX domains, through which they modulate the activity of the target proteins associated with these domains, such as polyP polymerases, P_i_ transporters, or P_i_-dependent plant transcription factors (Wild et al., 2016; Puga et al., 2017). A variety of InsPP isomers exist in cells (Figure 3), but it is unclear whether different isomers assume different signaling functions, and might represent an inositol pyrophosphate “code” (Azevedo and Saiardi, 2017; Gerasimaite et al., 2017; Shears, 2017). Available data do not provide a consistent picture. Whereas studies on the transcriptional phosphate starvation response (PHO pathway) concluded that phosphate starvation is signaled through increasing the 1-InsP_7_ concentration (Lee et al., 2007, 2008), later studies on the VTC complex indicated that phosphate starvation is signaled by a decline of 5-InsP_7_ (Lonetti et al., 2011; Wild et al., 2016; Gerasimaite et al., 2017). In contrast, studies on the mammalian P_i_ exporter XPR1 provided strong evidence for its exclusive regulation through 1,5-InsP_8_ (Li et al., 2020). Further work will be necessary to clarify whether the transcriptional phosphate starvation response and the transport and storage of P_i_ through SPX-containing proteins are indeed regulated by different inositol pyrophosphate isomers or whether these isomers might serve as signals to integrate different physiological parameters with P_i_ signaling (Azevedo and Saiardi, 2017; Shears, 2017).

SPX domains and InsPPs were found in many eukaryotes, and mutations affecting them give corresponding phenotypes (Secco et al., 2012a; Wilson et al., 2013; Shears, 2015; Wild et al., 2016; Puga et al., 2017). Since, in addition, SPX domains are frequently associated with proteins that mediate the uptake, export, storage, or foraging for P_i_, we can postulate an evolutionarily conserved signaling pathway in which cytosolic P_i_ is measured, translated into a corresponding change of InsPPs, and thereby communicated to a multitude of SPX-containing proteins (Figure 2). We term this pathway INPHORS (intracellular phosphate reception and signaling). We postulate that InsPPs address these proteins in a coordinated fashion in order to maintain cytosolic P_i_ in a suitable range. Then, we must expect that SPX domains bound to inositol pyrophosphates can have either activating or inactivating effects, depending on the proteins that they associate with. Since the levels of inositol pyrophosphates increase with the level of available P_i_, proteins exporting P_i_, either across the plasma membrane or into intracellular storage places such as the acidocalcisome-like vacuole, should be activated by inositol pyrophosphate-bound SPX. Likewise, we should expect that the activation of the transcriptional phosphate starvation response, the PHO pathway, should be repressed by InsPPs. This scheme is partially consistent with existing data because the SPX-containing polyP polymerase VTC is activated through the interaction between its SPX domains and the inositol pyrophosphate 5-InsP_7_ (Auesukaree et al., 2005; Lonetti et al., 2011; Wild et al., 2016; Gerasimaite et al., 2017) and because the low-affinity P_i_ importers Pho87 and Pho90 are inhibited by their respective SPX domains (Hürlimann et al., 2009). In contrast, conflicting data exist on other SPX proteins. The SPX-containing kinase Pho85/80/81, which represses the PHO pathway, was reported to be silenced by the inositol pyrophosphate 1-InsP_7_ (Lee et al., 2007). Furthermore, patch-clamp experiments suggested that the vacuolar P_i_ exporter Pho91 is activated by 5-InsP_7_ (Potapenko et al., 2018), although we would expect the inverse. It has also been suggested that inorganic pyrophosphate (PPi) can stimulate Pho91 (Potapenko et al., 2019). An argument against this hypothesis is that stimulation was observed with a high concentration of this compound, which is toxic in yeast and maintained at very low cytosolic levels by the essential enzyme Ipp1 (Serrano-Bueno et al., 2013). Further analyses will be needed in order to elucidate the reasons for the inconsistencies mentioned above and/or to allow us to revise our working hypothesis.

## The P_i_ Transporters of Yeast

Yeast cells can take up P_i_ through five transporters (Pho84, Pho87, Pho89, Pho90, and Pho91). The deletion of all five transporters together is lethal, but the quintuple mutant can be rescued by the overexpression of any one of these five importers (Wykoff and O’Shea, 2001) or by the overexpression of *GIT1*, a glycerophosphoinositol permease that also transports P_i_ and glycerol-3-phosphate (Wykoff and O’Shea, 2001; Popova et al., 2010). A further transporter in the plasma membrane, Syg1, is presumed to export P_i_ due to its homology to the mammalian P_i_ exporter XPR1 (Giovannini et al., 2013).

### High-Affinity P_i_ Transporters: Pho84 and Pho89

Pho84 and Pho89 are high-affinity transporters since they permit the uptake of P_i_ from low-P_i_ media (<0.2 mM) with a *K*_m_ of 8–40 μM (Bun-ya et al., 1991; Wykoff and O’Shea, 2001; Auesukaree et al., 2003; Levy et al., 2011). While Pho84 is a H^+^ symporter with a pH optimum in the acidic range (Bun-ya et al., 1991; Pérez-Sampietro et al., 2016), Pho89 is active at alkaline pH and driven by Na^+^ (Martinez and Persson, 1998; Zvyagilskaya et al., 2008; Serra-Cardona et al., 2014). In addition to P_i_, Pho84 serves also for the low-affinity co-transport of manganese ions (Jensen et al., 2003) and for the import of selenite (Lazard et al., 2010). Pho84 is highly expressed at intermediate concentrations of P_i_ (<0.5 mM), but becomes transferred to the vacuole for degradation in the absence of P_i_ or at high excess of P_i_ (Petersson et al., 1999; Pratt et al., 2004; Lundh et al., 2009). Pho84 allows the cells to exploit very low P_i_ concentrations, and their growth arrests only below a threshold of 5 μM. In standard P_i_-rich media, Pho84 is required for the maintenance of normal polyP levels (Hürlimann et al., 2007) and for repressing transcription through the PHO pathway (Wykoff et al., 2007). However, constitutive activation of the PHO pathway in cells lacking Pho84 does not reflect a major contribution of this high-affinity transporter to P_i_ uptake under P_i_-replete conditions. It is rather the consequence of the downregulation of the low-affinity transporters, which results from the deletion of PHO84 due to feedback regulation (Wykoff et al., 2007; Vardi et al., 2013). This feedback regulation creates a bistable, Spl2-dependent switch, which dedicates cells either to the P_i_-starved regulatory state (downregulating the low-affinity P_i_ transporters and inducing the PHO pathway) or to the P_i_-replete state (stabilizing the low-affinity transporters and repressing the PHO pathway). 14-3-3 proteins (Bmh1 and Bmh2, which also interact with the inositol hexakisphosphate kinase Kcs1) were proposed to influence this commitment (Teunissen et al., 2017), but the nature of their influence has not yet been resolved. The decision between the two states may depend on small differences in the cytosolic P_i_ concentration, i.e., on the degree to which cells experience P_i_ limitation, or on stochastic fluctuations. The bistable switch allows a fraction of the cells in a population to stably maintain their commitment to the phosphate starvation response for multiple generations—even after phosphate has been replenished. This can be seen as a potential advantage because these cells would be best prepared for a sudden drop in P_i_ availability and facilitate the survival of the population under such conditions.

As mentioned above, Pho84 has also been proposed to act as a P_i_ transceptor, which could provide an alternative explanation how it represses the PHO pathway under phosphate-replete conditions. Obtaining clear evidence for an activity as a transceptor is difficult because it requires maintaining signaling while suppressing the transporter function entirely. In the case of Pho84, one caveat is that the substrates that were used to induce putative transceptor signaling in the absence of transport, such as glycerol-3-phosphate, might be hydrolyzed extracellularly by yeast and the released P_i_ taken up by Pho84 (Popova et al., 2010). Also, transport-deficient point mutants retain low-capacity transport, which, in a P_i_-starved, non-dividing cell, may nevertheless suffice to satisfy the needs. Finally, the complex feedback regulation mentioned above complicates the interpretation of the experiments that suggested Pho84 as a transceptor (Giots et al., 2003; Mouillon and Persson, 2005; Popova et al., 2010; Samyn et al., 2012).

### SPX-Containing Low-Affinity P_i_ Transporters: Pho87 and Pho90

Pho87 and Pho90 are two low-affinity plasma membrane transporters which mediate the uptake of P_i_ from the environment with *K*_m_ values of 200–800 μM (Tamai et al., 1985; Wykoff and O’Shea, 2001; Auesukaree et al., 2003; Giots et al., 2003; Pinson et al., 2004; Hürlimann et al., 2007, 2009). They belong to the divalent anion:Na^+^ symporter (DASS) family. Both Pho87 and Pho90 harbor an SPX domain at their N-terminus. Removal of this SPX domain leads to phenotypes suggesting an enhanced, unrestricted flux of P_i_ across these transporters: up to an eightfold increased P_i_ uptake activity, higher total phosphate, and higher polyP content. Cells expressing Pho90 without an SPX domain also become sensitive to high P_i_ concentrations in the media, and they show an enhanced leakage of cellular P_i_ on low-P_i_ media (Hürlimann et al., 2009). Together, these observations indicate that the SPX domain has a restrictive function on Pho87 and Pho90, which is necessary to close these transporters at excessively high and low concentrations of P_i_.

The transcription of the *PHO87* and *PHO90* genes is independent of P_i_ availability, but the transporters are targeted to the vacuole and degraded in response to P_i_ limitation (Auesukaree et al., 2003; Wykoff et al., 2007; Hürlimann et al., 2009; Ghillebert et al., 2011). This targeting requires their SPX domain and, for Pho87, also Spl2. But why do cells have low-affinity transporters in addition to a high-affinity system? When the low-affinity importers are ablated, the cells compensate by expressing more Pho84, and this largely rescues their P_i_ uptake activity. While this demonstrates that a high-affinity transporter can substitute for the low-affinity system, even under high-P_i_ conditions (Wykoff and O’Shea, 2001; Pinson et al., 2004; Ghillebert et al., 2011), it brings up the question what benefit a cell draws from expressing low-affinity transporters when it has high-affinity transporters available. An advantage becomes apparent in situations where Pi gradually becomes limiting, for example in a culture that is exhausting the available Pi as it grows. At an intermediate P_i_ concentration, both high- and low-affinity transporters are expressed. The decrease in P_i_ availability can be detected earlier and over a much larger range of external P_i_ concentrations when the cells use low-affinity transporters as long as P_i_ is abundant and begin to express high-affinity transporters only once P_i_ becomes scarcer. In an environment of gradually depleting P_i_, this provides for a longer transition phase from P_i_-replete conditions to full P_i_ starvation, giving the cells much more time to adapt and prepare for P_i_ starvation (Levy et al., 2011). This leads to enhanced recovery from growth arrest once P_i_ becomes available again.

### SPX-Controlled Vacuolar P_i_ Transporter: Pho91

Pho91 is a low-affinity P_i_ transporter. Like the low-affinity P_i_ transporters Pho90 and Pho87, it belongs to the DASS family and its expression is independent of P_i_ supply (Auesukaree et al., 2003). However, a green fluorescent protein (GFP) fusion of Pho91 is localized to vacuoles (Hürlimann et al., 2007). When overexpressed in a mutant lacking all other P_i_ importers, Pho91 can nevertheless support the growth and P_i_ uptake of cells with a *K*_m_ of around 200 μM (Wykoff and O’Shea, 2001). Initially, this was taken as an indication that it operates at the plasma membrane. However, yeast cells can take up nutrients also by other routes—as shown for magnesium, which can be acquired by endocytic transfer to the vacuole lumen and subsequent transport across the vacuolar membrane into the cytosol (Klompmaker et al., 2017). Such an uptake route *via* vacuoles might also allow P_i_ acquisition through Pho91. In further support of a function at the vacuolar membrane, the ablation of Pho91 leads to an overaccumulation of phosphate in vacuoles, whereas its overexpression depletes this vacuolar pool. This led to the proposal that Pho91 transfers P_i_ from vacuoles to the cytosol (Hürlimann et al., 2007).

This view could be confirmed by electrophysiological analysis of Pho91 and of its homologs from rice OsSPX-MFS3 and *Trypanosoma brucei* TbPho91 (Wang et al., 2015; Potapenko et al., 2018), where a Na^+^-dependent transport of P_i_ into the cytosol with *K*_m_ values of 1–10 mM could be demonstrated. The transport activity is highly pH-dependent, as demonstrated for OsSPX-MFS3 (Wang et al., 2015). Whereas net charge transport occurs at neutral pH, the protein does not mediate significant P_i_ transport under these conditions. P_i_ is only transported in the presence of a pH gradient, when the extra-cytosolic face of the protein is exposed to acidic pH. This pH dependency corresponds to the natural condition under which these Pho91-like transporters operate because the lumen of the vacuoles in which they reside is much more acidic than the cytosol. In the absence of a pH gradient, OsSPX-MFS3 mediates P_i_ efflux from the cytosol (Wang et al., 2015). For TbPho91 and Pho91, it was shown that charge transport depends on their SPX domain and on 5-InsP_7_. Other inositol polyphosphates, such as 1-InsP_7_ or InsP_6_, do not activate the channel (Potapenko et al., 2018). However, these experiments have been conducted in the absence of a proton gradient across the membrane. Thus, while they demonstrate a regulatory impact of SPX and 5-InsP_7_ on the transporter, their effects on P_i_ transport under a pH gradient remain to be determined.

Overall, these observations suggest that Pho91 transports P_i_ from the vacuole into the cytosol (Hürlimann et al., 2007), which might be necessary to allow the degradation of vacuolar polyP. It might then be a critical element of the system to buffer cytosolic P_i_ *via* polyP (Neef and Kladde, 2003; Thomas and O’Shea, 2005). However, direct evidence for an impact of Pho91 on cytosolic P_i_ and a coherent model for the functioning of a vacuolar, polyP-based P_i_ buffer is still missing.

### The SPX-Containing Putative P_i_ Exporter Syg1

Syg1 was identified as a genetic suppressor of defects in pheromone signaling and predicted to be on the plasma membrane (Spain et al., 1995). Syg1, the mammalian transporter XPR1, and the related PHO1 from *Arabidopsis* share around 30% of sequence identity and some common features, such as an N-terminal SPX and a C-terminal EXS (for ERD1/XPR1/SYG1) domain. The function of the EXS domain is still unknown. Although the transport activity for Syg1 itself has not yet been shown, its human homolog XPR1 does facilitate P_i_ export across the plasma membrane (Giovannini et al., 2013; Wilson et al., 2019; Li et al., 2020). Export depends on its SPX domain and on 1,5-InsP_8_. Thus, Syg1 likely acts as a P_i_ exporter in yeast.

## Polyphosphate Metabolism and Phosphate Homeostasis

Inorganic polyphosphate is a polymer of dozens to hundreds of orthophosphate (P_i_) linked by energy-rich phosphoric anhydride bonds. PolyP efficiently chelates ions such as Ca^2+^, Mg^2+^, and Mn^2+^. Yeast cells can accumulate up to a quarter of their dry weight in the form of polyphosphate (Langen and Liss, 1958), located mostly in their acidocalcisome-like vacuole (Saito et al., 2005). Minor amounts have also been associated with other organelles, such as mitochondria, the ER, or the periplasmic space (Pestov et al., 2004; Lichko L. et al., 2006; Kulakovskaya et al., 2010). The sequestered polyP is not an immobile aggregate. Instead, it appears to be highly mobilizable (Wiame, 1947; Urech et al., 1978). PolyP is necessary to rapidly buffer changes in the cytosolic phosphate levels that can result from sudden changes in P_i_ availability or consumption (Neef and Kladde, 2003; Thomas and O’Shea, 2005). The polyP in the acidocalcisomes of other organisms can also be mobilized, for example upon osmotic challenges or changes in nutrient supply (Docampo and Huang, 2016). The accumulation of polyP has a strong impact on cytosolic P_i_ homeostasis and should hence be carefully controlled by the cell (Desfougères et al., 2016). However, it remains a major unsolved question how the synthesis and degradation of polyP are coordinated to achieve this goal. While we have some insights into the synthesis of polyP, it remains an enigma how the cells can store polyP in a compartment that contains considerable polyphosphatase activities and how the conversion of polyP back into cytosolic P_i_ is regulated (Gerasimaite and Mayer, 2016).

### PolyP Synthesis by the VTC Complex

The VTC proteins form complexes which all contain Vtc1, a 14-kDa integral membrane protein that spans the membrane three times, and Vtc4, the catalytically active subunit that synthesizes polyP from nucleotide triphosphates (Müller et al., 2002, 2003; Hothorn et al., 2009). Catalytic activity requires Mn^2+^, which is located in the active site. All VTC proteins have a transmembrane region similar to Vtc1. In contrast to Vtc1, the other VTC proteins contain an additional SPX domain at the N-terminus and a hydrophilic central domain, both facing the cytosolic side of the membrane (Müller et al., 2003). The central domain accommodates the catalytic center in Vtc4, whereas it is assumed to have only regulatory, non-catalytic function in Vtc2, Vtc3, and Vtc5 (Hothorn et al., 2009; Desfougères et al., 2016). VTC complexes exist in different isoforms, which contain Vtc1 and Vtc4, plus either Vtc2 or Vtc3. Vtc5 can associate with VTC to increase its activity, but VTC functions at a lower basal activity without it (Desfougères et al., 2016). Vtc1/2/4 is mainly localized in the ER, whereas Vtc1/3/4 concentrates on the vacuoles, when the cells are cultivated in P_i_-replete media. Under P_i_ limitation, both complexes localize to the vacuoles (Hothorn et al., 2009).

VTC acts not only as a polyP polymerase but at the same time as a polyP translocase. It couples the synthesis of polyP at the cytosolic face of the membrane with its translocation into the lumen of the organelle (Gerasimaite et al., 2014). Continued synthesis of polyP by VTC requires the electrochemical gradient across the vacuolar membrane, which is established by V-ATPase. This gradient is assumed to provide the driving force translocating the negatively charged polyP chain (Wurst et al., 1995; Gerasimaite et al., 2014).

The activity of VTC can be assayed on isolated yeast vacuoles (Gerasimaite et al., 2014). This *in vitro* system allowed the discovery of the regulation of SPX domains through inositol pyrophosphates (Wild et al., 2016). While SPX domains bind various inositol polyphosphates and pyrophosphates with *K*_d_ values in the low micromolar or even sub-micromolar range (Wild et al., 2016), these different isomers show strikingly different agonist properties on VTC. Only InsPPs stimulate the enzyme at low micromolar concentrations, with 5-InsP_7_ being the isomer that appears to regulate VTC *in vivo* (Gerasimaite et al., 2017). Since InsP_7_ increases when cells are in P_i_-replete conditions and decreases under P_i_ starvation (Lonetti et al., 2011; Wild et al., 2016), VTC should synthesize polyP when cytosolic P_i_ is sufficiently high, but it should be switched off when this parameter is too low. This reflects the *in vivo* situation because cells accumulate polyP stocks when sufficient P_i_ is still available and they deplete this stock under P_i_ starvation or upon a transient excessive consumption of P_i_ during the S-phase (Langen and Liss, 1960; Bru et al., 2016). That VTC must be carefully controlled by the cells is also suggested by the fact that it has a major impact on cytosolic P_i_. Inappropriate overactivation of VTC can lead to P_i_ depletion from the cytosol and activate the PHO starvation pathway even on P_i_-rich media, whereas its silencing can suppress the PHO pathway on media with limiting P_i_ (Desfougères et al., 2016). PolyP storage is a major function of yeast vacuoles, which is probably the reason why polyP production also activates the vacuolar membrane fusion machinery. The resulting fusion of several vacuoles together increases the volume of the compartment, thereby accommodating the need for increasing storage space in a rapid and efficient manner (Müller et al., 2002; Desfougères et al., 2016).

### Polyphosphatases

#### The Exopolyphosphatase Ppx1

Ppx1 is a member of the DHH phosphoesterase superfamily, together with h-prune, a mammalian exopolyphosphatase (Tammenkoski et al., 2008). Ppx1 is a soluble enzyme which hydrolyzes polyP to release P_i_ and PP_i_ (Wurst and Kornberg, 1994). Hydrolysis is processive, i.e., the enzyme does not leave the polyP chain after having hydrolyzed its terminal P_i_ residue. Ppx1 hydrolyzes polyP chains as short as three P_i_ residues, but cannot degrade PP_i_ and ATP. The enzyme is metal-dependent, with a preference for Mg^2+^, and active from pH 5.5 to 9 (Tammenkoski et al., 2007).

Ppx1 is expressed irrespective of P_i_ availability (Ogawa et al., 2000). Initial studies ascribed it to the cytosol, but exopolyphosphatase activities in the plasma membrane and mitochondrial fractions were also related to Ppx1 (Lichko et al., 2003; Lichko L.P. et al., 2006). High-throughput localization studies through GFP fusion proteins suggested localization in the cytosol, but also some enrichment in the nucleus (Huh et al., 2003). In any case, Ppx1 appears to be localized outside the vacuoles, where the major polyP stores are kept. In line with this, its deletion affects neither the chain length nor the abundance of polyP in the cell (Lonetti et al., 2011). It was hence proposed that Ppx1 might counteract an the accumulation of polyP in the cytosol, which is toxic for cells (Gerasimaite et al., 2014). A further attractive possibility is that Ppx1 might trim polyP from proteins that are covalently modified with this polymer (Azevedo et al., 2015, 2018, 2019).

#### The Vacuolar Endopolyphosphatases Ppn1 and Ppn2

In its active, homo-tetrameric state, Ppn1 is a non-processive endopolyphosphatase which preferentially hydrolyzes long polyP in the midst of the chain rather than at its terminal phosphate residues. Depending on the conditions, however, it was also reported to have exopolyphosphatase activity *in vitro* (Kumble and Kornberg, 1996; Andreeva et al., 2015). Ppn1 activity requires Mn^2+^ or Mg^2+^. The enzyme can hydrolyze polyP down to P_i_ and tripolyphosphate, whereas pyrophosphate and ATP are potent inhibitors (Kumble and Kornberg, 1996). PPN1 gene expression is induced under P_i_ limitation, i.e., when vacuolar polyP pools are consumed (Kumble and Kornberg, 1996). PPN1 knockouts retain polyP in similar amounts as wild types, but their polyP is of higher chain length.

Ppn2 is a member of the phospoprotein phosphatase (PPP) superfamily of metallophosphatases that resides in the vacuolar lumen. The enzyme depends on Zn^2+^ and exclusively shows endopolyphosphatase activity (Gerasimaite and Mayer, 2017). Ppn1 and Ppn2 together constitute the major part of the vacuolar polyphosphatase activity. They are necessary to mobilize polyP stores under P_i_ starvation. In their absence, the cells accumulate polyP of excessively high chain length and they virtually show no short-chain polyP anymore.

## Phosphate “Recycling”

When P_i_ becomes limiting, the cells use the PHO pathway to induce first the high-affinity transporter Pho84. They secrete phosphatases to recover P_i_ from external sources and induce VTC expression to maximize their polyP stores (Ogawa et al., 2000; Springer et al., 2003; Thomas and O’Shea, 2005; Wykoff et al., 2007; Vardi et al., 2014). When the cells really starve for P_i_, the polyP stores are hydrolyzed (Langen and Liss, 1958; Sethuraman et al., 2001; Neef and Kladde, 2003; Thomas and O’Shea, 2005; Gerasimaite and Mayer, 2017). Using this strategy might be helpful to allow redifferentiation and ordered transition into the quiescent state (the G_0_ phase of the cell cycle), in which cells arrest growth and become more resistant to stresses (De Virgilio, 2011). This important transition often coincides with the induction of autophagy, which transfers large amounts of cytosolic material, including RNA and organelles, into vacuoles for degradation (De Virgilio, 2011). Their hydrolysis provides the cells with a source of degradable nucleotides and phospholipids. Profound P_i_ starvation induces the expression of enzymes that release P_i_ from these internal molecules. It appears likely that this represents a “last resort” because it makes little sense for a cell to deplete its pools of nucleotides or phospholipids, which are indispensable for the next cell division, unless it is the only means to survive. In line with this, cells in which these recycling pathways have been ablated show poor long-term survival on P_i_-free media (Xu et al., 2013).

Gde1 is a glycerophosphocholine phosphodiesterase which hydrolyzes glycerophosphocholine to glycerol-3-phosphate and choline (Fisher et al., 2005). Glycerol-3-phosphate can either be channeled into the synthesis of new phospholipids, into glycolysis, or it can be hydrolyzed by the glycerol-3-phosphatases Gpp1 and Gpp2 (Patton-Vogt, 2007). All these pathways effectively lead to the recycling of internal P_i_. They are of sufficiently high capacity to allow the cells to grow on glycerophosphocholine as the sole source of phosphate. That the recycling activity of Gde1 may be relevant to the maintenance of cytosolic P_i_ homeostasis is suggested by the fact that GDE1 gene expression is regulated by the PHO pathway and strongly induced in low-P_i_ conditions (Ogawa et al., 2000; Almaguer et al., 2004). Furthermore, Gde1 carries an N-terminal SPX domain, which is expected to subject the enzyme to regulation by the INPHORS pathway. However, the regulatory role of this SPX domain has not yet been experimentally confirmed. Interestingly, Gde1 shares its glycerophosphodiesterase domain with Pho81, a key regulator of the PHO pathway. Whether the glycerophosphodiesterase domain of Pho81 is catalytically active is also unknown.

Phm8 has originally been identified as a lysophosphatidic acid phosphatase (Reddy et al., 2008). Its expression is strongly induced by P_i_ starvation (Ogawa et al., 2000). Under P_i_ starvation, yeast cells show a significant reduction in lysophosphatidic acid, and this reduction has been ascribed to Phm8 (Reddy et al., 2008). The enzyme might thus participate in the recycling of phosphate from degraded phospholipids. Phm8 has also been identified as a nucleotide monophosphate phosphatase (Xu et al., 2013). The enzyme allows liberating P_i_ from a wide variety of nucleoside monophosphates. In its absence, cells cannot survive P_i_ starvation for prolonged periods of time, underscoring the relevance of its P_i_ recycling activities in this situation.

The repressible “alkaline” phosphatase Pho8 (Kaneko et al., 1982) shows maximal activity on artificial chromogenic substrates, such as *p*-nitrophenyl phosphate, at alkaline pH. However, the enzyme is membrane-anchored and resides within the vacuole, which is an acidic compartment with a pH ranging from 5 to 6. At this pH, Pho8 exhibits maximal activity toward phosphoserine and phosphothreonine, and it has therefore been proposed that it may serve to dephosphorylate peptides (Donella-Deana et al., 1993), which are imported into vacuoles through autophagy. The expression of the enzyme is induced upon P_i_ starvation through the PHO pathway (Kaneko et al., 1985). Pho8 can also dephosphorylate fructose-2,6-bisphosphate, which is generated in the cytosol, but the physiological relevance of this activity has remained unexplored, and it remains unclear how fructose-2,6-bisphosphate could be translocated into vacuoles, where Pho8 is located (Plankert et al., 1991). Pho8 may also participate in the recuperation of P_i_ from NAD^+^. Upon P_i_ starvation, part of the nicotinamide nucleotide pool may be converted into nicotinamide riboside, liberating P_i_ (Lu and Lin, 2011). This conversion requires Pho8 and the vacuolar nucleoside transporter Fun26. Yeast cells with a constitutively activated PHO pathway show increased production and secretion of nicotinamide riboside.

## Elements of the INPHORS Pathway

### The PIPP5 Kinase Vip1

Vip1 belongs to a conserved family of diphosphoinositol pentakisphosphate kinases (PPIP5Ks) (Randall et al., 2019). These enzymes contain both a kinase and a histidine acid phosphatase domain, which compete with each other (Mulugu et al., 2007). Their enzymatic and structural properties have mainly been uncovered through studies of the mammalian enzymes (Wang et al., 2012; Weaver et al., 2013; Gu et al., 2017; Nair et al., 2018; Randall et al., 2019), but the essential features could be confirmed for the plant and yeast members of the family (Pöhlmann et al., 2014; Dong et al., 2019; Zhu et al., 2019). The kinase domain phosphorylates InsP_6_ and 5-InsP_7_ to generate 1-InsP_7_ and 1,5-InsP_8_, respectively, whereby 5-InsP_7_ appears to be the preferred substrate over InsP_6_ (Wang et al., 2012; Weaver et al., 2013). The simultaneous presence of competing kinase and phosphatase domains, which, in addition, appear to be coordinated by allosteric effects (Yousaf et al., 2018), has important consequences. It can translate small changes in the concentration of the substrates into much larger changes of product concentration, i.e., it can amplify the response in a signaling cascade. Under suitable conditions (for PIPP5Ks, when only the phosphatase but not the kinase domain is substrate-limited), it can also make the net kinase activity quite insensitive to changes in substrate concentrations (Gu et al., 2017). This property would allow the cell to modulate 5-InsP_7_ without an immediate impact on the level of 1,5-InsP_8_, providing an important prerequisite to use these inositol pyrophosphates to communicate different cellular parameters, which might then be integrated by proteins that can “read” several different inositol pyrophosphates, such as SPX domains.

Importantly, the phosphatase activity of PIPP5Ks is inhibitable by P_i_ in the low millimolar range—a concentration range that is commonly found in the cytosol—and their kinase activity is boosted by an increasing ATP concentration (Gu et al., 2017; Zhu et al., 2019). Since cellular ATP concentration diminishes with cellular P_i_ availability (Boer et al., 2003), P_i_ limitation in the cytosol should tip the equilibrium between the kinase and phosphatase activities in favor of dephosphorylation, decreasing the pools of 1,5-InsP_8_ and 1-InsP_7_. PIPP5Ks might thus provide an important link between the P_i_ concentration and the corresponding changes in the inositol pyrophosphate levels. However, whether PIPP5Ks represent the critical P_i_ sensor is not yet clear. Also, the physiological implications of the sensitivity of PIPP5K to phosphoinositides, such as PI(4,5)P_2_ (Nair et al., 2018), are not yet understood.

### The InsP_6_ Kinase Kcs1

Kcs1 is a member of the inositol hexakisphosphate kinase family and phosphorylates the phosphate on carbon 5 of InsP_6_ or Ins(1,3,4,5,6)P_5_, creating 5-InsP_7_ or 5PP-InsP_4_, respectively (Saiardi et al., 1999, 2000; Figure 3). The physiological relevance of 5PP-InsP_4_ is uncertain because it accumulates to appreciable levels only in a mutant lacking IPK1, which cannot convert Ins(1,3,4,5,6)P_5_ into InsP_6_ and, hence, does not offer InsP_6_, which is the far more abundant substrate of Kcs1 in a wild-type cell, where it probably outcompetes Ins(1,3,4,5,6)P_5_. Kcs1 shares a PxxxDxKxG motif in its catalytic site with other members of the family. The ablation of Kcs1 activity leads to the constitutive activation of the PHO transcription pathway even on high-P_i_ media, to an overaccumulation of P_i_ and ATP, and to a complete absence of polyP synthesis (Auesukaree et al., 2005; Lonetti et al., 2011; Szijgyarto et al., 2011; Wild et al., 2016). In contrast, KCS1 overexpression represses the PHO pathway even under P_i_ limitation, where it would normally be maximally active (Auesukaree et al., 2005). The inactivation of this enzyme thus produces the effects that we would expect if its product 5-InsP_7_ was a negative regulator of the phosphate starvation response. Erroneous activation of the phosphate starvation program in a kcs1Δ cell under high-P_i_ conditions should then lead to an overaccumulation of P_i_. Although the synthesis of polyP normally occurs under high-P_i_ conditions (Langen and Liss, 1958), this requires the activation of the VTC complex by 5-InsP_7_, which is not present in kcs1Δ cells (Lonetti et al., 2011; Wild et al., 2016). Therefore, kcs1Δ mutants cannot accumulate polyP.

Kcs1 has a *K*_m_ for InsP_6_ of 0.6 μM and an exceptionally high *K*_m_ for ATP of around 1 mM (Saiardi et al., 1999). By consequence, its activity should be sensitive to the fluctuations of cytosolic ATP concentrations that normally occur in living yeast cells (Saiardi et al., 1999). This may be one reason why mutants in nucleotide metabolism that show constitutively lower cellular ATP levels—and thereby probably lower production of 5-InsP_7_ and 1,5-InsP_8_—lead to a constitutive activation of the PHO pathway (Choi et al., 2017). The cellular ATP concentration in wild-type cells declines as a result of P_i_ limitation (Boer et al., 2010). This opens the possibility that P_i_ limitation might be translated into a reduction of 5-InsP_7_ and 1,5-InsP_8_ through a reduced ATP production.

### The Inositol Pyrophosphatases Ddp1 and Siw14

Inositol pyrophosphates can be turned over by two dedicated phosphatases, which selectively hydrolyze the phosphoric anhydride bonds in these molecules. They have considerable influence on the steady-state levels of inositol pyrophosphates, but it is unknown whether their activity is constitutive or regulated.

Ddp1 belongs to the Nudix hydrolase family. In cells lacking the *DDP1* gene, the abundance of InsP_7_ increases up to sixfold (Lonetti et al., 2011; Steidle et al., 2016). Ddp1 displays di-phosphoinositol polyphosphate hydrolase activity (Safrany et al., 1999). It also exhibits di-adenosine polyphosphate hydrolase activity (Cartwright and McLennan, 1999; Safrany et al., 1999), which is of unclear physiological significance in yeast. Ddp1 can hydrolyze polyP (Lonetti et al., 2011). However, the enzyme is localizing to the cytosol and the nucleus, i.e., out of reach of the major polyP reserves, which are localized in the vacuole (Saito et al., 2005). This makes it unlikely that Ddp1 influences P_i_ homeostasis through polyP turnover. In line with this, its deletion has no significant impact on the polyP pool (Lonetti et al., 2011). Cells lacking Ddp1 show a 20% reduction of polyP rather than an increase. This reduction might be a secondary consequence of the deregulation of the INPHORS pathway and of an altered cellular P_i_ homeostasis due to the influence of Ddp1 on inositol pyrophosphates. The polyphosphatase activity of Ddp1 might, however, serve to modify the polyphosphorylation of proteins in the nucleus (Azevedo et al., 2015).

Siw14 belongs to the atypical dual-specificity phosphatase family and hydrolyzes the ß-phosphate of 5-InsP_7_ with high specificity (Steidle et al., 2016; Wang et al., 2018). Mutants lacking *SIW14* display a sixfold increase in InsP_7_ content. This effect is synergistic with the simultaneous deletion of *DDP1* and the double mutants display a 20-fold increase in InsP_7_ (Steidle et al., 2016). Overexpression of *SIW14* depletes the InsP_7_ pool.

### Inositol Pyrophosphate Receptors: SPX Domains

SPX domains are found in all eukaryotic kingdoms and share common sequence features, most notably clusters of positively charged amino acids. These conserved arginine and lysine residues cluster in a surface patch which forms a high-affinity (sub-micromolar) binding site for inositol polyphosphates and pyrophosphates. Purified SPX domains show a relatively low discrimination in binding different inositol polyphosphates or pyrophosphate isomers, and their affinity decreases in parallel to the net charge of the compound (InsP_8_ > InsP_7_ > InsP_6_). In marked contrast to the low selectivity in binding, the agonist properties of different isomers vary considerably. The VTC complex, for example, is strongly activated by 1,5-InsP_8_, but very poorly by 5-PPP-InsP_5_, which carries the same number of phosphate groups (Gerasimaite et al., 2017). Likewise, 5-InsP_7_ activates VTC, whereas InsP_6_ has virtually no effect. Thus, despite the extremely high charge density of the inositol polyphosphates and their similar binding affinities, the SPX domain must decode precise structural features of the ligands and translate them into different degrees of activation.

Neither the binding affinity nor the EC_50_ values of inositol pyrophosphate isomers seem to faithfully reflect their physiological relevance for controlling an SPX domain. This could be illustrated with VTC, which has an EC_50_ for 5-InsP_7_ that is similar to that of 1-InsP_7_ and is 20-fold higher than that of 1,5-InsP_8_. Nevertheless, genetic ablation of the enzyme synthesizing 1-InsP_7_ and 1,5-InsP_8_ (Vip1) has no significant impact on polyP synthesis *in vivo*. The ablation of 5-InsP_7_ synthesis (Kcs1), in contrast, eliminates polyP synthesis, strongly suggesting this isomer as the relevant controller of the SPX domains of VTC *in vivo*. Whether 5-InsP_7_ is the principal regulator also for the other SPX-controlled proteins in the cell is an important issue that remains to be explored. The mammalian P_i_ exporter XPR1 provides an important example in this respect because this SPX-controlled transporter is opened by 1,5-InsP_8_ rather than by 1-InsP_7_ or 5-InsP_7_ (Li et al., 2020).

### Spl2

Spl2 interacts with the SPX domains of Pho90 and Pho87 and restrains the flux of P_i_ through Pho90 (Wykoff et al., 2007; Hürlimann et al., 2009). It is transcribed at a basal constitutive level, but it becomes further induced through the PHO pathway upon P_i_ limitation (Ogawa et al., 1995; Flick and Thorner, 1998). Together with the PHO81 gene, SPL2 was originally identified as a multicopy suppressor of the growth defect resulting from the ablation of the phospholipase C Plc1 (Flick and Thorner, 1998). Plc1 hydrolyzes phosphatidylinositol 4,5-biphosphate into 1,2-diacylglycerol and inositol 1,4,5-triphosphate, which is the precursor for the synthesis of inositol pyrophosphates (Saiardi et al., 2000). The genetic interactions of Spl2, Pho81, and Plc1 can thus be rationalized based on the premise that low cytosolic P_i_ is signaled by low levels of inositol pyrophosphates. The absence of Plc1 activity impairs the synthesis of inositol pyrophosphates and induces the phosphate starvation response (Auesukaree et al., 2005). When such cells are cultivated on P_i_-rich standard yeast media, one should expect that they behave incorrectly, i.e., they are maximizing their efforts for P_i_ uptake although this nutrient is abundant. A resulting excessive P_i_ concentration in the cytosol is the likely reason for their growth defect. This inappropriate regulation can be mitigated by the overexpression of Spl2, which limits P_i_ transporter activity (Hürlimann et al., 2009), or by the overexpression of Pho81 (Creasy et al., 1993; Ogawa et al., 1995), which inactivates the transcriptional phosphate starvation response. This sets cells back into the high-P_i_ mode, which then corresponds again to the P_i_-rich conditions of standard media, which had been used for these experiments (Flick and Thorner, 1998).

Upon P_i_ starvation, the low-affinity phosphate transporters Pho87 and Pho90 are endocytosed and targeted to the vacuole. For Pho87 and Pho90, this process depends on their SPX domains. Although the SPX domains of both transporters interact with Spl2, Spl2 is only necessary for the vacuolar targeting of Pho87, which occurs rapidly upon phosphate limitation. The degradation of Pho90, which occurs only after prolonged P_i_ limitation, is independent of Spl2 (Ghillebert et al., 2011; Pérez-Sampietro et al., 2016). Thus, Spl2 may allow the cells to differentially degrade their low-affinity transporters as a function of declining P_i_ availability.

The deletion of *SPL2* does not produce a striking growth phenotype on P_i_-rich standard yeast media (Flick and Thorner, 1998), but it results in an exacerbated sensitivity to selenite, probably due to the resulting hyperactivation of the low-affinity transporter Pho90, which is involved in selenite toxicity (Lazard et al., 2010).

## The PHO Pathway: a Transcriptional Phosphate Starvation Response

The PHO pathway is a transcriptional response pathway that regulates the expression of a wide variety of genes (Ogawa et al., 2000), many of which have been proven beneficial or essential for growth and survival under P_i_ limitation. The PHO pathway has been studied extensively, providing us with detailed insights into its regulation and with numerous tools to manipulate it. A key element is the nuclear Pho80/85/81 kinase, which is inactivated through its Pho81 regulatory subunit when P_i_ becomes limiting (Schneider et al., 1994). The kinase subunit Pho85 phosphorylates the transcription factor Pho4 and thereby shifts its localization toward the cytosol (Kaffman et al., 1994; O’Neill et al., 1996). Pho4 localization is hence defined through an equilibrium of Pho85-controlled import and export across the nuclear membrane. The removal of Pho4 from the nucleus under P_i_-replete conditions represses the expressions of genes controlled by the PHO pathway. In the following, we describe the key regulatory factors, but we will discuss only the most important PHO-regulated target genes.

### The PHO Regulon: Phosphate-Responsive Genes (the PHO Genes)

The transcription factors Pho4 and Pho2 co-regulate the expressions of dozens of genes (Bun-ya et al., 1991; Oshima, 1997; Ogawa et al., 2000). Among these genes are the P_i_ transporters Pho84 (Bun-ya et al., 1991), Pho89 (Martinez and Persson, 1998), and the ER protein Pho86, which is involved in the targeting and exit of Pho84 from the ER (Yompakdee et al., 1996). PHO genes also encode several phosphatases (Persson et al., 2003): the repressible secreted acid phosphatase Pho5 and the cell wall-associated acid phosphatases Pho11 and Pho12 (Toh-e and Kakimoto, 1975; Arima et al., 1983; Bostian et al., 1983), which liberate P_i_ from phosphoester substrates; the vacuolar “alkaline” phosphatase Pho8 (Kaneko et al., 1985); and the glycerophosphocholine (GroPCho) phosphodiesterase Gde1 (Ogawa et al., 2000). All genes for the synthesis and degradation of vacuolar polyphosphate (*VTC1* through *VTC5*, *PPN1*, and *PPN2*) are also controlled by the PHO pathway (Ogawa et al., 2000).

The genes of some regulators of the PHO pathway are themselves transcribed under the control of this pathway, such as *PHO81* and *SPL2* (Wykoff et al., 2007; Hürlimann et al., 2009; Ghillebert et al., 2011). As described above, this generates the possibility of positive and negative feedback loops, which can lead to heterogeneity in a yeast population, e.g., with a fraction of cells remaining stably committed to the P_i_ starvation program even under P_i_-replete conditions (Wykoff et al., 2007; Vardi et al., 2013, 2014; Estill et al., 2015).

Besides the genes mentioned above, the PHO pathway regulates numerous other genes, among them many open reading frames of unknown function, for which the relationship to P_i_ homeostasis has not yet been understood (Ogawa et al., 2000). A similar complexity of P_i_-regulated transcription has been found in other fungi, e.g., in *Cryptococcus neoformans* and *Schizosaccaromyces pombe*, where the number of PHO genes has been determined to be in the range of 130–160 (Henry et al., 2011; Carter-O’Connell et al., 2012; Lev and Djordjevic, 2018). They suggest links of the PHO signaling pathway to cellular transport, carbohydrate and lipid metabolism, and the responses to stress and to chemicals.

### Regulation of PHO Genes During P_i_ Limitation and Serious P_i_ Starvation

The transition of *S. cerevisiae* into phosphate starvation is a complex process in which the cells show a graded adaptation to the declining P_i_ availability and temporally separate waves of gene induction. When P_i_ gradually becomes limiting, the activity of Pho85 kinase declines, leading first to a partial dephosphorylation of the Pho4 transcription factor. The partially dephosphorylated Pho4 induces only part of the PHO genes, such as the high-affinity transporter Pho84. Others, such as the secreted acid phosphatase Pho5, follow only upon more profound P_i_ starvation, leading to the inactivation of Pho85 kinase and the full dephosphorylation of Pho4 (O’Neill et al., 1996; Springer et al., 2003; Thomas and O’Shea, 2005). This establishes a graded response, which first maximizes phosphate acquisition and, upon more serious starvation, activates the recycling of P_i_ from internal resources.

The activation of the PHO pathway is also influenced by a metabolic intermediate of purine biosynthesis, 5-aminoimidazole-4-carboxamide ribonucleotide (AICAR) (Pinson et al., 2009). AICAR binds Pho2 and Pho4 *in vitro* and promotes their interaction *in vivo*, suggesting a direct effect on these transcription factors. Since AICAR also transcriptionally regulates purine biosynthesis, it may coordinate phosphate acquisition with nucleotide synthesis. Nucleotide synthesis is a major sink for P_i_, and such coordination might be useful for this reason. Importantly, the activation of the PHO pathway through AICAR does not coincide with a major accumulation of Pho4 in the nucleus, in striking contrast to the induction through Pho85/80/81. AICAR thus represents an alternative pathway to activate the transcription of PHO pathway genes.

Detailed analyses of the temporal pattern of PHO gene activation revealed further features that stabilize the transcriptional response and keep the cells from erroneously self-inactivating the PHO pathway (Vardi et al., 2014). This is relevant under moderate P_i_ limitation, where such self-inactivation might occur, because the measures by which the cells seek to improve P_i_ acquisition may completely rectify the initial decline in cytosolic P_i_ that triggered the starvation response, thereby leading to an oscillation of the PHO pathway activation and inactivation. This cannot happen upon complete depletion of P_i_ from the media. A decisive difference is thus whether the cells experience only moderate P_i_ limitation or profound P_i_ starvation. Upon moderate limitation of P_i_, a first wave of transcription induces a subset of genes: for the high-affinity P_i_ importer Pho84; Pho86, which is involved in its biogenesis; the secreted acid phosphatases Pho12 and Pho11 (Vardi et al., 2014); the genes for the VTC complex, which depletes cytosolic P_i_ and transfers it as polyP into vacuoles; Spl2, which shuts down the low-affinity P_i_ transporters; and Pho81, which reduces Pho85 kinase activity and promotes Pho4-dependent transcription. This wave of gene induction enhances global P_i_ absorption and storage by the cells and, at the same time, establishes a positive feedback loop that stabilizes the induction of Pho4-dependent gene expression (Wykoff et al., 2007; Vardi et al., 2013, 2014). This feedback loop maintains the activation of the PHO pathway for several generations, even after the cells have been transferred back into high-P_i_ media.

Cells experiencing profound P_i_ starvation (no P_i_ in the medium) reveal other features. They induce the same set of immediately activated genes as cells in intermediate P_i_. However, due to the absence of P_i_ in the media, the induction of these genes cannot suffice to stabilize their cytosolic P_i_. The more pronounced and stable decline in cytosolic P_i_ is believed to trigger a second wave of gene inductions 2 h after the first wave (Vardi et al., 2014). This second wave additionally activates genes for the intracellular recuperation of P_i_ from nucleotides, lipids, and other substrates (Phm8 and Pho8); the high-affinity P_i_ importer Pho89; and the secreted phosphatase Pho5. This second wave of transcription coincides with the induction of the environmental stress response and a decline in the growth rate. The partial dephosphorylation of the transcription factor Pho4 may be the trigger for the first transcriptional wave (Springer et al., 2003; Vardi et al., 2014), whereas full dephosphorylation of Pho4 might trigger the second wave of induction. Furthermore, the promoter regions of the genes induced in these two waves show characteristic differences that may support this differential activation through their impact on the chromatin structure (Lam et al., 2008), which is also influenced by inositol polyphosphates (Steger et al., 2003).

Upon serious P_i_ starvation, the cells finally arrest their cell cycle after two further divisions, and they enter into the quiescent G_0_ phase of the cell cycle. Pho85/80/81 kinase influences the entry and exit into quiescence in at least two ways: It phosphorylates and thereby inhibits the Rim15 kinase, which is a key factor for G_0_ entry (Wanke et al., 2005). Under P_i_-replete conditions, Pho85/80/81 is active and phosphorylates Rim15, which then concentrates in the cytosol, where it is sequestered from its nuclear targets that initiate the G_0_ program. The residue that becomes phosphorylated on Rim15 is the same as the one phosphorylated through the TOR pathway, suggesting that P_i_ availability is integrated with information about the availability of other nutrients, such as amino acids, at the level of Rim15. Pho85/80/81 also phosphorylates and thereby destabilizes the cyclin Cln3, which is necessary for the exit from G_0_ (Menoyo et al., 2013). Both activities may synergize to favor an entry into G_0_ when P_i_ and, hence, Pho85/80/81 activities are low.

## Elements of the PHO Pathway

### Pho85, Pho80, and the Regulation of Transcription Through Pho4 and Pho2

Pho4 is a transcription factor which is expressed independently of the P_i_ concentration in the medium (Yoshida et al., 1989a). It carries a transactivation domain and a basic helix–loop–helix motif. Pho2 is a transcriptional activator bearing a homeobox (Bürglin, 1988). It interacts with Pho4 (controlling the PHO pathway), but also with a variety of transcription factors, such as Swi5 (controlling mating type switching) and Bas1 (controlling purine and histidine biosynthesis) (Bhoite et al., 2002). Its interaction with Pho4 increases the affinity for Pho4 binding sites in the promoter region of the PHO genes and allows them to recruit the general transcription machinery (Shao et al., 1996; Magbanua et al., 1997a, b).

Pho85 is a cyclin-dependent kinase subunit that associates with at least 10 different cyclins to regulate a wide spectrum of target proteins involved in many cellular processes, such as cell cycle control, storage and metabolism of carbohydrates, amino acid metabolism, and calcium signaling (Toh-E et al., 1988; Toh-E and Nishizawa, 2001; Huang et al., 2007). The cyclins confer target specificity to Pho85. Virtually all Pho85 effects that are directly relevant to phosphate homeostasis are mediated through its association with the cyclin Pho80. Pho80, and by consequence also the Pho85/80 kinase, are localized in the nucleus. This localization is independent of P_i_ availability (Hirst et al., 1994; Schneider et al., 1994). Pho85/80 is constitutively associated with Pho81, which inhibits its kinase activity under P_i_ limitation. Nuclear Pho85/80/81 phosphorylates the transcription factor Pho4 at five sites (O’Neill et al., 1996; Komeili and O’Shea, 1999). One of these phosphorylations blocks the association of Pho4 with the transcription factor Pho2; others promote the interaction of Pho4 with the nuclear export receptor Msn5 (Kaffman et al., 1998a) and impair its interaction with the nuclear import receptor Pse1 (Kaffman et al., 1998b). By means of these phosphorylations, Pho85/80 hinders the transcription of PHO genes in two ways: by impairing the interaction of Pho4 with the second necessary transcription factor Pho2 and by favoring the export of Pho4 into the cytosol.

### Pho81

Pho81 is a cyclin-dependent kinase inhibitor (CKI) and the major regulator of Pho85/80 kinase and the PHO pathway (Schneider et al., 1994). Pho81 binds the Pho80 subunit of the Pho85/80 complex independently of Pi availability, but it inhibits the kinase only in low-P_i_ conditions (Schneider et al., 1994; Ogawa et al., 1995; O’Neill et al., 1996; Huang et al., 2001). Pho81 can also regulate the Pho85/Pcl7 cyclin-CDK complex in a P_i_-dependent manner, which controls glycogen metabolism (Lee et al., 2000). PHO81 gene expression is induced by Pho4 upon P_i_ limitation (Bun-ya et al., 1991; Creasy et al., 1993, 1996; Ogawa et al., 2000), and Pho81 itself is also a substrate for Pho85/80 kinase (Knight et al., 2004; Waters et al., 2004). This creates a possibility for feedback regulation.

Pho81 is a large protein of almost 1,200 amino acids, which can be dissected into three functional domains (Ogawa et al., 1995). An N-terminal SPX domain and a C-terminal part, both of which influence the degree to which P_i_ starvation can induce the PHO pathway. The central part of the protein contains six ankyrin repeats. Part of these repeats and an adjacent region form a 141-amino acid piece that is sufficient to maintain some P_i_-dependent regulation of the PHO pathway *in vivo*. The resulting inducibility is limited, however, because this central domain yields only a fivefold induction of the *PHO5* gene on P_i_-free media, and this only upon overexpression, whereas full-length Pho81, expressed at normal levels from its native promoter, yields an almost 200-fold induction (Ogawa et al., 1995). This central region was later trimmed further to remove the remaining ankyrin repeats, resulting in an 80-amino acid region, termed “minimum domain.” Also, the minimum domain allows only a less than 10-fold induction of PHO5 when overexpressed (Huang et al., 2001). Nevertheless, the minimum domain was ascribed a key function in the regulation of the PHO pathway through Pho81 (Lee et al., 2007, 2008). That the other parts of Pho81 may as well play a crucial role in regulation is suggested by the observation that the minimum domain is not sufficient to allow Pho85/80 to regulate the stress response pathway in a P_i_-dependent manner (Swinnen et al., 2005), which full-length Pho81 does. Furthermore, several mutations leading to the constitutive activation of the PHO pathway lie in the N-terminal part of Pho81 (Creasy et al., 1993; Ogawa et al., 1995), underlining the physiological relevance of this region.

It has been reported that Pho81 requires the PIPP5K Vip1 and its product 1-InsP_7_ to inhibit Pho85/80 kinase and that InsP_7_ shows a corresponding increase under P_i_ starvation (Lee et al., 2007). Although this model has been widely accepted, several observations cannot easily be reconciled with it: Firstly, the InsP_7_ concentration actually decreases with decreasing P_i_ availability and Vip1 makes only a minor contribution to the InsP_7_ pool in rich media (Lonetti et al., 2011; Steidle et al., 2016; Wild et al., 2016). Secondly, the inactivation of the enzymes Plc1, Ipk1, and Arg82, which ablates the synthesis of 1-InsP_7_ and its precursor InsP_6_, inhibits Pho85/80 (Auesukaree et al., 2005) instead of showing the activation that one should expect if InsP_7_ inhibited the enzyme. Thirdly, vip1Δ mutants can also activate the PHO pathway, though with a moderate delay relative to wild-type cells (Choi et al., 2017). Thus, it is currently unclear how the PHO pathway is regulated through inositol pyrophosphates and the availability of P_i_.

### Control of the PHO Pathway Through Other Parameters Than Pho4 Activation

Besides phosphate limitation, several other conditions can activate PHO genes. Potassium starvation upregulates the *PHO* genes *PHO84*, *PHO5*, and *SPL2* using the *PHO* signaling pathway (Anemaet and van Heusden, 2014; Canadell et al., 2015). Also, growth on acidic or alkaline pH, or the activation of calcineurin, can trigger the PHO pathway (Causton et al., 2001; Serrano et al., 2002; Ruiz et al., 2003; Luan and Li, 2004). The cell cycle activates PHO genes transiently and in a cyclic manner through Pho4 (Spellman et al., 1998), but also through the transcription factor Fkh2 (Pondugula et al., 2009; Korber and Barbaric, 2014; Bru et al., 2016).

While some activation of the PHO pathway may be related to secondary effects of the tested conditions on cytosolic P_i_ concentration, it is clear that Pho4/Pho2 are not the only transcription factors inducing PHO gene expression. These genes are embedded into a complex network of transcriptional control, which engages many other players, including the SAGA complex (Nishimura et al., 1999), the SWI-SNF complex (Gregory et al., 1999), the nucleosome spacing factor Ino80 (Barbaric et al., 2007), the arginine methyl transferase Hmt1 (Chia et al., 2018), or the transcriptional activator Crz1 (Serra-Cardona et al., 2014). These factors can either directly activate transcription or they may regulate promoter activity through the chromatin structure (Steger et al., 2003; Lam et al., 2008; Korber and Barbaric, 2014).

Yeast cells also perform pervasive transcription, which produces non-coding RNAs (Xu et al., 2009). Antisense transcripts for *PHO5* and *PHO84* mediate an exosome-dependent reduction in the abundance of messenger RNA (mRNA) from these two genes (Camblong et al., 2007) and affect chromatin remodeling during the activation of *PHO5* transcription (Uhler et al., 2007). On the *PHO84* promoter, it recruits the histone deacetylase Hda1, which downregulates transcription (Camblong et al., 2007). At the *KCS1* locus, antisense and intragenic transcripts are produced, and their production depends on the transcription factor Pho4 and on P_i_ starvation (Nishizawa et al., 2008). These transcripts lead to the synthesis of truncated Kcs1, which is presumed to show lower activity.

Phosphate-dependent regulation of gene expression appears to act also on steps subsequent to the initiation of transcription. For example, Pho92 regulates the degradation of Pho4 mRNA by binding to its 3′-UTR in a P_i_-dependent manner (Kang et al., 2014). The nonsense-mediated decay pathway is probably involved in this mRNA degradation reaction through Pop2 and the Ccr4-NOT complex.

### Outlook

P_i_ homeostasis is an essential and complex aspect of metabolism. Even a simple unicellular organism such as yeast dedicates a significant percentage of its only 6,000 genes on it, underlining its fundamental importance. Many components involved in the P_i_ homeostasis in yeast are conserved in other organisms, suggesting that there are common approaches to the problem. Others appear functionally conserved, but realized in a quite organism-specific manner. For example, P_i_-dependent transcriptional control is a widespread phenomenon which is conserved in that it employs SPX domains and InsPPs, and we expect that the way in which SPX domains interact with their target proteins may reveal common features. However, in other organisms, the mediators transmitting this control to the transcription factors can differ from the PHO pathway components acting in yeast. Work in a variety of different model organisms will thus contribute essential information necessary to grasp the full spectrum of strategies underlying P_i_ homeostasis.

Yeast cells are a prime model to study phosphate homeostasis in a eukaryotic cell system because many of the proteins immediately implicated in stabilizing cytosolic P_i_ are known and important elements of the signaling cascades regulating their expression and activity have begun to emerge. A major challenge lies in the high degree of redundancy that cells use in order to regulate their internal phosphate store. In order to study the regulatory mechanisms behind P_i_ homeostasis, it will therefore be necessary to establish reduced and simplified systems, both *in vivo* and *in vitro*, which will allow isolating individual aspects of the complex regulatory network and exploring them without too much interference by redundant mechanisms. The potent and convenient methods for manipulating the yeast genome provide the necessary tools to tackle this task.

## Author Contributions

Both authors wrote all parts of the review together.

## Conflict of Interest

The authors declare that the research was conducted in the absence of any commercial or financial relationships that could be construed as a potential conflict of interest.
